# Three New Species and Three New Records of Arthoniaceae (Ascomycota, Arthoniales) from China

**DOI:** 10.3390/jof12010042

**Published:** 2026-01-06

**Authors:** Chengyue Hao, Shuhua Jiang, Zefeng Jia

**Affiliations:** 1 College of Agriculture and Biology, Liaocheng University, Liaocheng 252059, China; 2 State Key Laboratory of Mycology, Institute of Microbiology, Chinese Academy of Sciences, Beijing 100101, China

**Keywords:** lichenized fungi, morphology, phylogeny, taxonomy

## Abstract

Based on morphological and phylogenetic analyses, during a study on the biodiversity of the lichenized fungi family Arthoniaceae in Yunnan, China, three new species of *Arthonia*, *Eremothecella*, and *Synarthonia* (*Arthonia yunnanensis* sp. nov., *A. pararubella* sp. nov., *Eremothecella pruinocarpa* sp. nov.) and three new Chinese records (*A. rubella*, *E. calamicola*, *Synarthonia inconspicua*) were reported in this present study. A phylogenetic analysis using maximum likelihood and Bayesian inference based on a two-gene dataset (mtSSU and RPB2) revealed that all these species form distinct independent clades. In addition, this study represents the first report of molecular sequences for the genus *Eremothecella*. Detailed descriptions, ecological and chemical characteristics, and illustrations for each species are provided.

## 1. Introduction

The family Arthoniaceae Rchb. (belonging to Ascomycota, Arthoniales) is one of the largest families of lichenized fungi, and was formally delineated by Reichenbach [1]. It exhibits two important morphological features: a reduction of the ascomatal borders and globose to clavate asci with a strongly thickened apical wall, and it is not monophyletic [2,3,4,5]. This family is mainly prominent in tropical regions with abundant corticolous and foliicolous species, and it constitutes a major component of the lichen flora across diverse forest types [6]. Including genera such as *Arthonia* Ach., *Eremothecella* Syd., and *Synarthonia* Müll. Arg., the family currently comprises 20 genera and approximately 750 species [7,8].

*Arthonia* (established by Acharius in 1806) remains one of the most understudied taxa even though it encompasses approximately 500 species in the world [9]. This genus is considered to be a heterogeneous group, according to Grube et al. [10]. The lichenized representatives of *Arthonia* are always crustose with irregular or stellate (rarely rounded) ascocarps and have hyaline to brownish, muriform, or transversely septate (often unequally) ascospores [11]. The genus (at least in its traditional sense) comprises all different life forms: lichens, non-lichenized fungi, lichen parasites, and parasymbionts [5].

The genus *Eremothecella*, established by Sydow [12], shows distinctive morphological characteristics, including dark brown to black ascomata with loose paraphysoids, globose asci frequently emerging through the ascomata surface as small warts, and applanate pycnidia producing elongated filiform conidia with multiple septa, which provide taxonomic separation from *Arthonia* [13,14]. The genus was merged into *Arthonia* by Santesson [15], but subsequent revisions by Sérusiaux [13] recognized the generic distinctness of *Eremothecella* and formally accepted four species within this classification: viz*. E. calamicola* Syd., *E*. *macrosperma* (Zahlbr). Sérus., *E*. *palmulacea* (Müll. Arg.) Sérus., and *E*. *variratae* (Aptroot & Sipman) Sérus. Lücking (2008) further affirmed its taxonomic autonomy based primarily on the presence of *Eremothecella*-type pycnidia [14]. Currently, ten species are recognized: eight foliicolous and two corticolous in this genus [16].

*Synarthonia* was established in 1891. This small genus was the subject of a detailed discussion and report by Van Den Broeck et al. [17]. It is characterized by solitary ascomata becoming mono- to pluri-carpocentral synascomata embedded in a slightly elevated to immersed pseudostroma, with a thin white thalline margin, and *Arthonia*-type asci producing transversely septate ascospores, with enlarged apical cell or muriform ascospores [18]. However, the phylogeny of *Synarthonia* was unclear, and the synonymy of *Reichlingia* Diederich & Scheid had been suggested, but Ertz et al. (2020) pointed out that *Synarthonia* can be placed in a well-supported monophyletic lineage [18,19].

During our ongoing studies on Arthoniaceae in China, specimens belonging to three genera—*Arthonia*, *Eremothecella*, and *Synarthonia*—were collected. Integrative taxonomic analyses based on combined phenotypic and molecular datasets were conducted [20]. For molecular phylogenetic analyses, the mitochondrial small subunit (mtSSU) and the RNA polymerase subunit II (RPB2) were used, following the methods described by Frisch et al. (2014) and Thiyagaraja et al. (2020) for Arthoniaceae [5,6]. Three new species and three new records of Arthoniaceae from China are reported here.

## 2. Materials and Methods

### 2.1. Morphological and Chemical Analyses

Specimens were collected from Yunnan Province, China, and were deposited in the Fungarium of the College of Life Sciences, Liaocheng University (LCUF). Freshly collected specimens were first subjected to DNA extraction or preserved at −20 °C. The extracted DNA was typically stored at −80 °C, and all specimens were ultimately deposited in the herbarium. An Olympus dissecting microscope (Olympus SZX16, Olympus Corporation, Tokyo, Japan) and an Olympus light microscope (Olympus BX53, Olympus Corporation, Tokyo, Japan) were used for morphological and anatomical studies. Measurements were taken from mature vertical sections of fruit bodies mounted in water to observe whether they contained crystals: the shape and size of the photobiont, the width of the hyphae, and the color, shape, and size of the asci, ascospores, pycnidia, and conidia. The amyloidity of ascospores was tested with Lugol’s solution. Spot tests with K (10% aqueous solution of potassium hydroxide), C (saturated solution of aqueous sodium hypochlorite), P (saturated solution of p-phenylenediamine in 95% ethylalcohol), and UV (fluorescence at 365 nm) were performed on the surface of the thallus [21]. The lichen substances were detected and identified using a UV light source and thin-layer chromatography (TLC) in solvent C [22].

### 2.2. DNA Extraction and PCR Sequencing

Genomic DNA was extracted from the ascomata and thallus of the specimens using a DNA secure Plant Kit (Tsingke, Beijing, China) according to the manufacturer’s instructions. The mtSSU and RPB2 regions were amplified using the primer pair mtSSU1/3R [23] and RPB2-7cF/11aR [24]. Polymerase Chain Reaction (PCR) cycling conditions were set to an initial denaturation at 95 °C for 5 min, followed by 35 cycles of denaturation at 95 °C for 45 s, annealing at 50 °C for 1 min, extension at 72 °C for 1.5 min, and a final extension at 72 °C for 10 min [25]. The PCR products were checked on 1% electrophoresis gels, stained with ethidium bromide, and sequenced by Tsingke Biotech Co., Ltd. (Beijing, China).

### 2.3. Phylogenetic Analyses

The new sequences were assembled via Geneious v.9.0.2 (Biomatters Ltd., Auckland, New Zealand) and potential sample contamination were confirmed by BLASTn searches on the NCBI website (http://www.ncbi.nlm.nih.gov/BLAST/, accessed on 17 April 2025). Then, high-similarity sequences of Arthoniaceae were selected for further analysis (Table 1), with *Melarthonis piceae* Frisch & G. Thor (belonging to Chrysotrichaceae, Arthoniales) as the outgroup [5]. The assembled sequences were aligned with the online version of MAFFT v.7 (https://mafft.cbrc.jp/alignment/server/, accessed on 17 April 2025). Ambiguously aligned regions were delimited using Gblocks v.0.91b with the least stringent selection [26]. Geneious v.9.0.2 was used to concatenate the mtSSU and RPB2 genes and produce a two-locus dataset. Maximum likelihood (ML) analysis and Bayesian inference (BI) were used to infer phylogenetic trees based on the concatenated mtSSU and RPB2 datasets. The maximum likelihood analysis was conducted with RAxML-HPC 2 on ACCESS v.8.2.12 employing a GTRGAMMA approximation with a rapid bootstrap analysis of 1000 replicates on the CIPRES Scientific gateway portal (http://www.phylo.org/portal2/, accessed on 17 April 2025) for verification [27,28]. For BI analysis, PartitionFinder 2 [29] was used to determine the best-fit model for each partition. Based on the results, we used the GTR+I+G model for mtSSU and the HKY+I+G model for RPB2. Bayesian Inference phylogenies were inferred using MrBayes v.3.2.7a under a partition model [30]. We ran 2 million generations, in which the initial 25% of sampled data was discarded as burn-in. Bayesian inference posterior probability (BIPP) equal to and above 95% was considered significant and shown at the nodes. Generated phylogenetic trees were visualized under Figtree v.1.4.4 and edited in Adobe Illustrator CC2019 software.

## 3. Results

### 3.1. Phylogenetic Results

**Figure 1:**
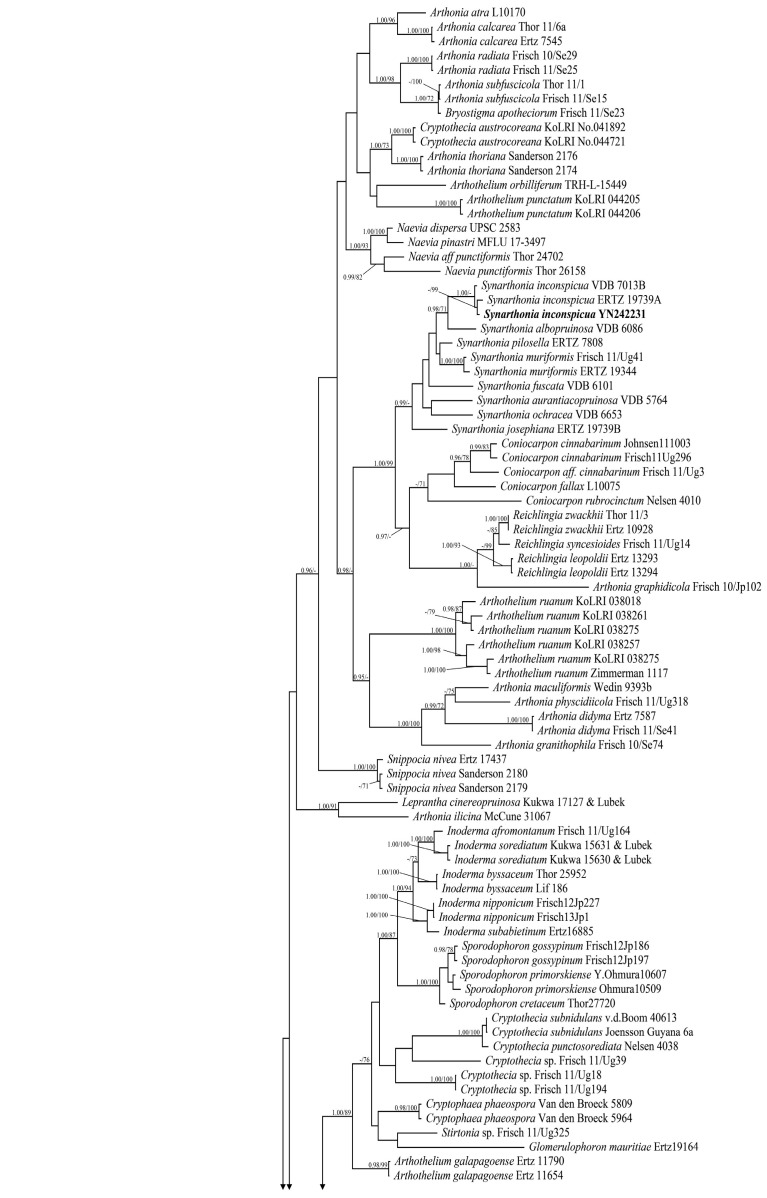

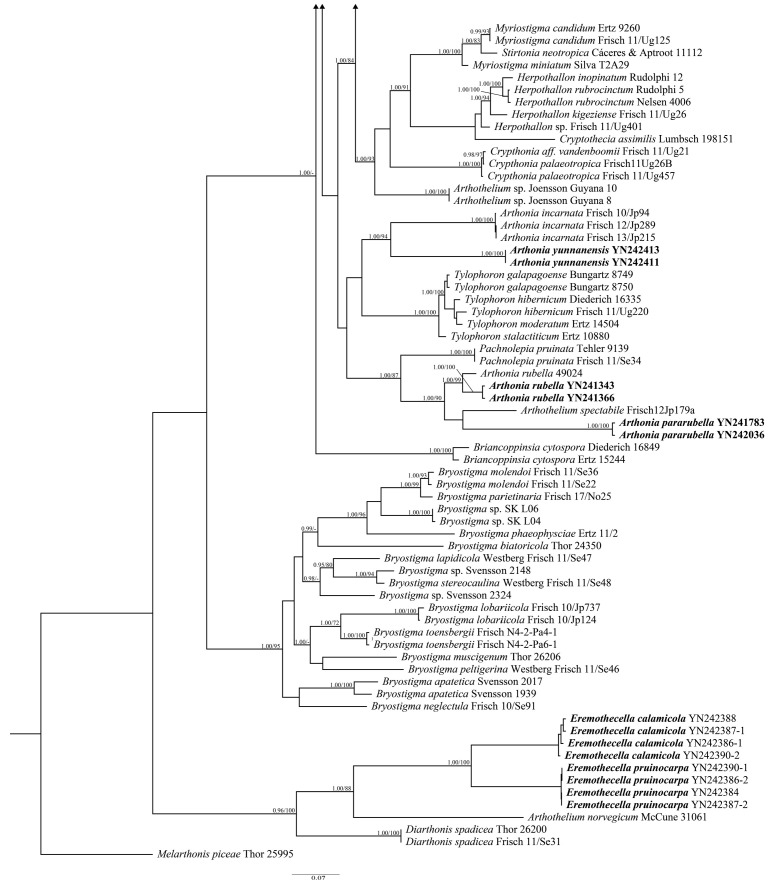
Phylogenetic tree of the family Arthoniaceae constructed from Bayesian analyses based on a two-gene dataset (mtSSU and RPB2). Support values are on the branches (posterior probability (PP)/bootstrap value (BS)). Bayesian inference posterior probabilities above 0.95 (Left) and maximum likelihood bootstrap support above 70% (Right) are shown at nodes. The new sequences in this study are in bold.

A total of 150 specimens comprising 227 DNA sequences (139 mtSSU sequences and 88 RPB2 sequences), including fifteen mtSSU sequences and eight RPB2 sequences that were newly generated were used in this study (Table 1). For the two-locus (mtSSU and RPB2) dataset taxon set, the concatenated alignment contained 1544 characters (787 for mtSSU and 757 for RPB2). The RAxML tree was constructed with a final ML optimization likelihood value of −27,812.975243. The parameters for the GTR+I+G model of combined mtSSU and RPB2 were as follows: estimated base frequencies: A = 0.290705, C = 0.231758, G = 0.228597, T = 0.248940; substitution rate AC = 1.017805, AG = 5.448594, AT = 1.720312, CG = 1.388442, CT = 5.952238, and GT = 1.000000. The resulting topology was similar for maximum likelihood (ML) and Bayesian analysis. Therefore, only the Bayesian tree is presented, with bootstrap support ≥70% for ML analysis and posterior probabilities ≥0.95 for Bayesian analysis (Figure 1).

In the phylogenetic tree (Figure 1), the main well-supported lineages of Arthoniaceae follow the results obtained by Frisch et al. (2014) and agree well with previous hypotheses on the polyphyletic origins of parasitic *Arthonia* [6]. Our phylogenetic results also support that *Eremothecella* forms an independent clade within the family Arthoniaceae based on the sequence of *Eremothecella pruinocarpa* (YN242390-1, YN242386-2, YN242384, YN242387-2) and *E. calamicola* (YN242388, YN242387-1, YN242386-1, YN242390-2), which were newly generated in this study. These are also the first reported molecular sequences of species in the genus *Eremothecella*.

### 3.2. Taxonomy

**Figure 2:**
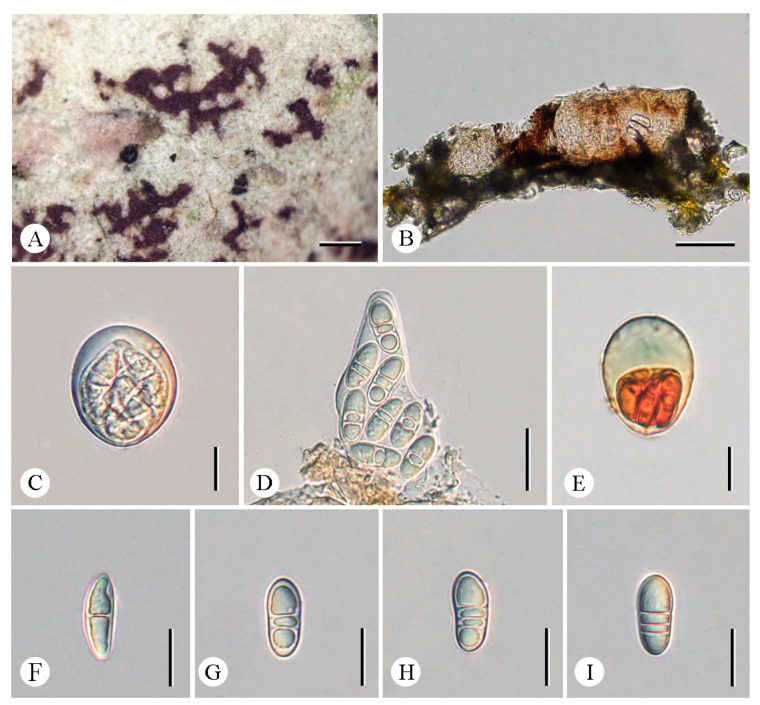
*Arthonia pararubella* (LCUF YN242036) (**A**) thallus with ascomata (**B**) apothecium section (**C**,**D**) asci, and with eight ascospores (**E**) asci in 10% KOH stained with Lugol’s solution (**F**–**I**) ascospores in water. Scale bars: 0.5 mm (**A**); 50 µm (**B**); 20 µm (**C**–**I**).

***Arthonia pararubella*** C.Y. Hao, S.H. Jiang, and Z.F. Jia, sp. nov. (Figure 2).

Fungal Name: FN 572511.

**Diagnosis.** The new species externally resembles *Arthonia rubella* (Fée) Nyl. but differs in the shorter ascospores and fewer septa.

**Type.** China. Yunnan Province: Mengla County, Xishuangbanna Tropic Botanical Garden, Chinese Academy of Sciences, Baihuayuan, 21°55′26″ N, 101°15′02″ E, alt. 560 m, on bark, 18 December 2024, C.Y. Hao (LCUF YN242036—holotype).

**Description. **Thallus greenish gray to whitish gray. Prothallus brownish-black line. Ascomata reddish-brown to blackening, innate, maculate to lirellate, sometimes branched or stellate, thinner at the branched ending, 0.2–1.65 × 0.05–0.2 mm. Excipulum carbonized, 21.5–30 μm thickness. Hymenium 67–77 µm tall, pale brown to brown, K/I+ blue. Asci clavate to subglobose, 47.5–66.5 × 28–44 µm ($\bar{X}$ = 55 × 35 μm), *Arthonia*-type, eight-spored, I+ orange-red, apical part thickened, usually with a distinct ocular chamber, K/I−. Ascospores oblong–ovoid, (2–)3-septate, upper and lower cells enlarged, ellipsoidal locules in immaturity, colorless, 25.5–29.5 × 10–13.5 μm ($\bar{X}$ = 28 × 12.5 μm), I+ orange-red.

**Chemistry.** Thallus K-, C-, KC-, UV-. No substances were detected by TLC.

**Ecology and distribution.** The new species is currently only known in China on the bark of trees in a tropical forest in Yunnan Province.

**Etymology.** The specific epithet *pararubella* refers to the similarity to *Arthonia rubella*.

**Additional specimens examined.** China. Yunnan Province: Jinghong City, Mengyang Town, Wild Elephant Valley, 22°09′58″ N, 100°51′49″ E, alt. 800 m, on bark, 16 December 2024, C.Y. Hao (LCUF YN241783).

**Notes.** *Arthonia pararubella* can be distinguished from similar species by its hyaline ascospores (25.5–29.5 × 10–13.5 μm) with two to three septa. *Arthonia rubella* is morphologically similar to this new species, but the former can be distinguished by longer ascospores with more septa (3–5-septate vs. (2–)3-septate; 25–43 × 10–15 μm vs. 25.5–29.5 × 10–13.5 μm) [31,32,33]. Further, both are far apart in the phylogenetic tree (Figure 1). Another similar species, *A. compensatula* Nyl. differs from this new species by its ascospores with more septa (4–5-septate vs. (2–)3-septate) and without amphicephalic [33,34].

**Figure 3:**
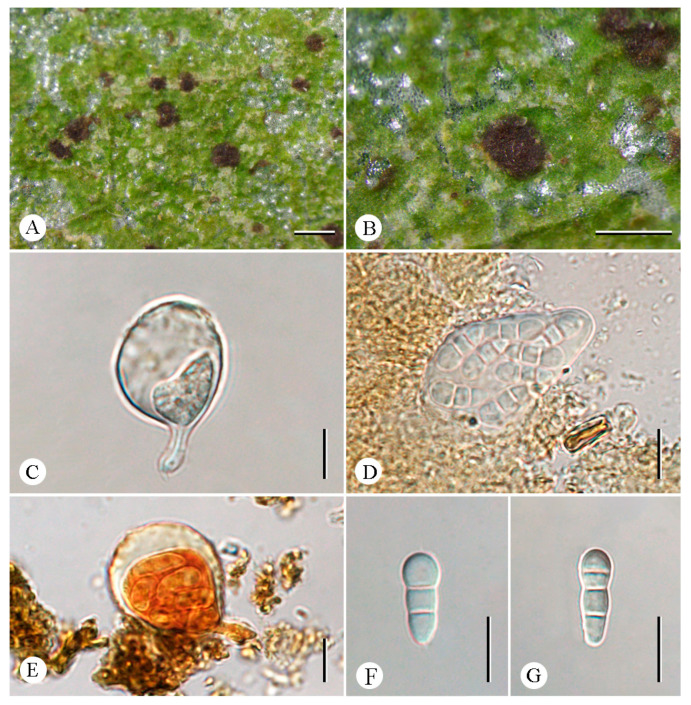
*Arthonia yunnanensis* (LCUF YN242413) (**A**,**B**) thallus with ascomata (**C**,**D**) asci, and with eight ascospores (**E**) asci in 10% KOH stained with Lugol’s solution (**F**,**G**) ascospores in water. Scale bars: 0.5 mm (**A**,**B**); 10 µm (**C**–**G**).

***Arthonia yunnanensis*** C.Y. Hao, S.H. Jiang, and Z.F. Jia, sp. nov. (Figure 3).

Fungal Name: FN 572510.

**Diagnosis.** The new species externally resembles *Arthonia incarnata* Kullh. ex Almq, but differs in the subglobose asci, color of the thallus, and that it lives on leaves.

**Type.** China. Yunnan Province: Mengla County, Xishuangbanna Tropic Botanical Garden, Chinese Academy of Sciences, lvshilin, 21°54′17″ N, 101°16′28″ E, alt. 625 m, on leaves, 18 December 2024, C.Y. Hao (LCUF YN242413—holotype).

**Description.*** *Thallus uncontinuous, yellowish green to green, thin, matte to weakly glossy. Prothallus absent. Photobiont trentepohlioid, cells globose to long elliptical. Ascomata dispersed, maculate, usually slightly irregularly roundish to short elliptical, adnate, flat to weakly convex, pale and often brownish to dark brown, 0.25–0.4 mm in diameter. Hymenium near hyaline to pale orange-brown, K/I+ blue. Asci subglobose, 27–32 × 18–23 μm ($\bar{X}$ = 29 × 20.5 μm), *Arthonia*-type, thin lateral walls, stipitate, eight-spored, usually with a distinct ocular chamber, K/I−. Ascospores clavate, 2(–3)-septate, usually with an enlarged distal cell, not curved, with slight constrictions at septa, colorless, 12.5–15.5 × 4.5–6.5 μm ($\bar{X}$ = 14 × 5.5 μm).

**Chemistry.** Thallus K-, C-, KC-, UV-. No substances were detected by TLC.

**Ecology and distribution.** The new species is currently only known in China on the leaves of trees in a tropical rainforest in Yunnan Province.

**Etymology.** The specific epithet *yunnanensis* refers to the type locality, Yunnan Province, Mengla County.

**Additional specimens examined.** China. Yunnan Province: Mengla County, Xishuangbanna Tropic Botanical Garden, Chinese Academy of Sciences, Greenstone Forest, 21°54′17″ N, 101°16′28″ E, alt. 625 m, on leaves, 18 December 2024, C.Y. Hao (LCUF YN242411).

**Notes.** *Arthonia yunnanensis*, the only species from southern China’s tropical region inhabiting leaves, is characterized by subglobose asci. It resembles *Arthonia incarnata*, *A. accolens* Stirt., and *A. lividula* Vain. in having 2-septate ascospores [4,15,35,36]. However, *A. incarnata* resembles the new species, having the same size ascospores (11.7–15.3 × 4.2–5.4 μm vs. 12.5–15.5 × 4.5–6.5 μm), but its thallus is often inapparent or visible as a whitish to pale olive-gray discoloration of bark; further, its asci are longer and narrower (40–50 × 14–20 μm vs. 27–32 × 18–23 μm) [4]. In the phylogenetic tree (Figure 1), although *A*. *yunnanensis* forms a sister clade to *A. incarnata*, the two clades are distinctly separated by a long genetic distance and exhibit substantial divergence. Therefore, *A*. *yunnanensis* as an independent lineage also supports its status as a species distinct from *A. incarnata*. *Arthonia accolens* can be distinguished by larger ascomata (often >0.5 mm diam.) [15,36]. *Arthonia lividula* differs from this new species in having shorter asci and smaller ascospores without constrictions at the septa (9–12 × 3–5 μm vs. 12.5–15.5 × 4.5–6.5 μm) [15,35]. Morphologically, *A*. *yunnanensis* was revealed as significantly different from all known species of *Arthonia* (see notes below), and therefore we describe this species here as a new species.

**Figure 4:**
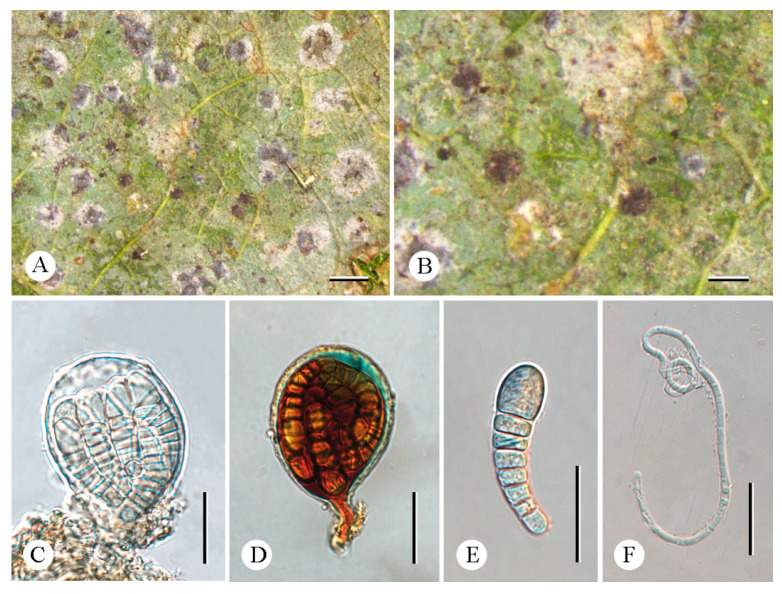
*Eremothecella pruinocarpa* (LCUF YN242390-1) (**A**) thallus, with ascomata (**B**) thallus, pycnidia (**C**) asci, and eight ascospores (**D**) asci in 10% KOH stained with Lugol’s solution (**E**) ascospores in water. (**F**) conidia in water. Scale bars: 1 mm (**A**); 0.5 mm (**B**); 20 µm (**C**–**F**). Fungal name: FN 572512.

***Eremothecella pruinocarpa*** C.Y. Hao, S.H. Jiang, and Z.F. Jia, sp. nov. (Figure 4).

**Diagnosis.** The new species externally resembles *Eremothecella ajaysinghii* Jagad. Ram & G.P. Sinha but differs in its fewer septa of ascospores.

**Type.** China. Yunnan Province: Jinghong City, tropical rainforests, 21°54′31″ N, 101°10′58″ E, alt. 635 m, on leaves, 17 December 2024, C.Y. Hao (LCUF YN242390-1—holotype).

**Description. **Thallus crustose, greenish gray, dispersed or continuous, matte. Prothallus absent. Photobiont trentepohlioid, cells in radiate plates, rectangular, ca. 11 × 4 μm. Ascomata rounded to irregular, dark brown to black, ± moderately to densely grayish pruinose, pruina sometimes increasingly dense along the margin, 0.2–1.15 mm diam. Asci obovate to globose, 45–60 × 36.5–45.5 μm ($\bar{X}$ = 54.5 × 41 μm), stipitate, eight-spored, K/I+ red, usually with a distinct ocular chamber, K/I+ blue. Ascospores colorless, clavate, curved, (6–)7(–9)-septate, enlarged distal cell, very slight constrictions at septa, 30–49 × 8–11.5 μm ($\bar{X}$ = 38 × 9.5 μm), I+ orange-red. Pycnidia numerous, ± rounded, irregular, black, 0.2–0.45 mm diam. Conidia colorless, filiform, multiseptate, 80–130 × 1.5–2 µm ($\bar{X}$ = 100 × 2 μm).

**Chemistry.** Thallus K-, C-, KC-, UV-. No substances were detected by TLC.

**Ecology and distribution.** The new species is currently only known in China on the leaves of trees in a tropical rainforest in Yunnan Province.

**Etymology.** The specific epithet *pruinocarpa* refers to the densely whitish gray ascomata.

**Additional specimens examined.** China. Yunnan Province: Jinghong City, tropical rainforests, 21°54′31″ N, 101°10′58″ E, alt. 635 m, on leaves, 17 December 2024 X. Li (LCUF YN242384; LCUF YN242386-2; LCUF YN242387-2).

**Notes.** *Eremothecella pruinocarpa* differs from similar species in its grayish pruinose ascomata and (6–)7(–9)-septate ascospores. *Eremothecella ajaysinghii* resembles the new species *E. pruinocarpa* in having grayish pruinose ascomata but differs in having ascospores with more septa [8–10(–11)-septate vs. (6–)7(–9)-septate] [16]. *Eremothecella macrosperma* is another similar species, particularly in ascospore size, but its ascomata are non-pruinose [15]. *Eremothecella cyaneoides* Lücking differs from this new species in having ascospores with fewer septa (3–5-septate vs. (6–)7(–9)-septate) [37]. Phylogenetically, four specimens of *Eremothecella pruinocarpa* form a strongly supported monophyletic clade, which also supports that it is a new species (Figure 1).

**Figure 5:**
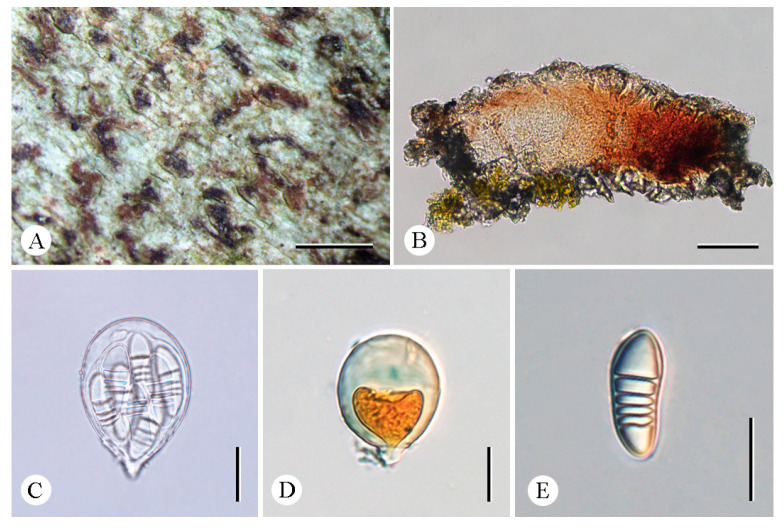
*Arthonia rubella* (LCUF YN241343) (**A**) thallus with ascomata (**B**) apothecium section (**C**) asci, and with eight ascospores (**D**) asci in 10% KOH stained with Lugol’s solution (**E**) ascospores in water. Scale bars: 1 mm (**A**); 50 µm (**B**); 20 µm (**C**–**E**).

***Arthonia rubella*** (Fée) Nyl. (Figure 5).

Fungal Name: FN 119459.

**Basionym.** *Graphis rubella* Fée, Essai Crypt. Exot. (Paris): 43 (1825) [1824].

**Description. **Thallus greenish gray to green, or whitish gray. Prothallus line brownish black. Photobiont trentepohlioid, cells globose. Ascomata reddish-brown and black, innate, maculate to lirellate, flexuous to irregularly branched, 0.25–1.2 mm long, disk occasionally opening. Asci clavate to subglobose, *Arthonia*-type, eight-spored, apical part thickened, usually with a distinct ocular chamber. Ascospores oblong–ovoid, (3–)4(–5)-septate, upper and the lower cell enlarged, colorless, 29–35 × 12–15 μm.

**Chemistry.** Thallus K-, C-, KC-, UV-. No substances were detected by TLC.

**Ecology and distribution.** Growing on exposed trees in tropical forests. Initially reported from America (as ‘*Graphis rubella*’) [31]. Newly reported in China.

**Additional specimens examined.** China. Yunnan Province: Puer City, Jingdong County, Jinping Town, Fork River Bridge, 24°37′30″ N, 100°45′22 ″ E, alt. 1320 m, on bark, 16 August 2024, C.Y. Hao (LCUF YN241343; LCUF YN241366).

**Notes.** The morphological and anatomical characteristics of this study align with *Arthonia rubella* from America as described by Fée (as ‘*Graphis rubella*’) [31]. The vast majority of records document this species on bark substrates in tropical South America [31,38]. It is closely related to *A. compensatula* in morphology, but the latter species has no amphicephalic ascospores [31,33,34]. It is also similar to *A. acanthotheciicola* Ertz & Common in ascospore morphology and septation, with both featuring enlarged upper and lower cells; however, the latter typically has smaller ascospores (14.5–17 × 6–6.5 µm) [39]. The newly generated *A. rubella* sequences from this study are clustered with the published sequences of the same species in the phylogenetic tree (Figure 1).

**Figure 6:**
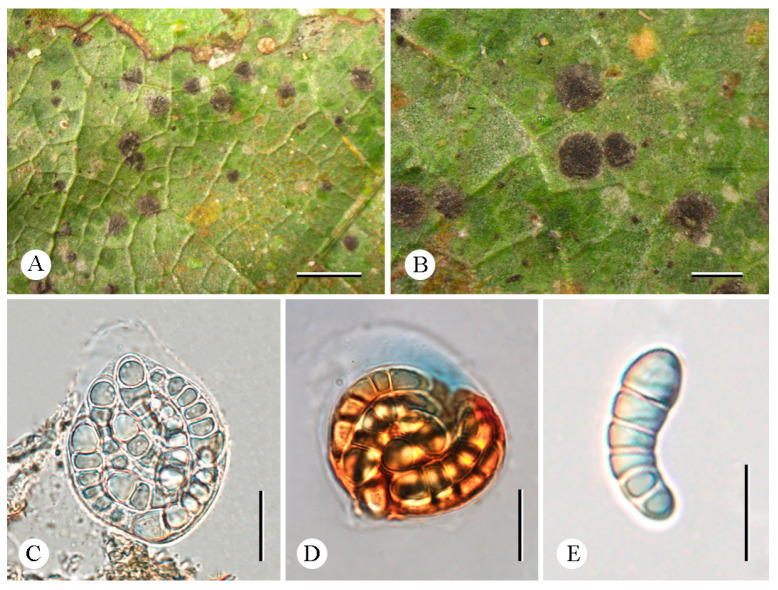
*Eremothecella calamicola* (LCUF YN242388) (**A**,**B**) thallus with ascomata (**C**) asci, with eight ascospores (**D**) asci in 10% KOH stained with Lugol’s solution (**E**) ascospores in water. Scale bars: 2 mm (**A**); 1 mm (**B**); 20 µm (**C**–**E**).

***Eremothecella calamicola*** Syd. (Figure 6).

Fungal Name: FN 145347.

**Description. **Thallus crustose, dispersed or continuous, smooth, green, thin, matte. Prothallus absent. Photobiont trentepohlioid, cells rectangular to irregular, ca. 9 × 5 μm. Ascomata rounded to irregular in outline, 0.3–1 mm diam., dark brown to blackish brown, non-pruinose. Asci obovate to globose, 37.5–52.5 × 33.5–40.5 µm, stipitate, eight-spored, usually with a distinct ocular chamber, K/I+ pale blue. Ascospores colorless, clavate, curved, 5–6-septate, enlarged distal cell, with very slight constrictions at septa, (28.3–)30.5–38 × 8.5–10.5 μm, I+ orange-red.

**Chemistry.** Thallus K-, C-, KC-, UV-. No substances were detected by TLC.

**Ecology and distribution.** Grows exposed trees in the shaded parts of evergreen forests. Previously reported from the Philippines [12,15], New Guinea [15], Mexico, Costa Rica, Panama, Guyana, French Guiana, Ecuador, Peru, Brazil [14], and India [16]. Newly reported in China.

**Additional specimens examined.** China. Yunnan Province: Jinghong City, tropical rainforests, 21°54′31″ N, 101°10′58″ E, alt. 635 m, on leaves, 17 December 2024, X. Li (LCUF YN242386-1; LCUF YN242387-1; LCUF YN242388); C.Y. Hao (LCUF YN242390-2).

**Notes.***Eremothecella calamicola* resembles *Arthonia palmulacea* (Müll. Arg.) R. Sant. but the latter has shorter ascospores with fewer septa (16–25 × 4–8 μm vs. (28.3–)30.5–38 × 8.5–10.5 μm; 3–5-septate vs. 5–6-septate) [15,16]. *Eremothecella variratae* is another similar species regarding ascospore size (28–42 × 7–10 µm vs. (28.3–)30.5–38 × 8.5–10.5 μm), but its ascomata often feature orange-yellow pruinose [15,16]. Another morphologically similar species, *E. nicobarica* Jagad. Ram and G. P. Sinha. can be distinguished by larger ascospores with more septa ((14–)15–17-septate vs. 5–6-septate; 60–72 × 10–13 µm vs. (28.3–)30.5–38 × 8.5–10.5 μm) [15,16]. In the phylogenetic tree, a well-supported monophyletic clade comprising the *E. calamicola* specimens is sister to the new species *E. pruinocarpa* (Figure 1).

**Figure 7:**
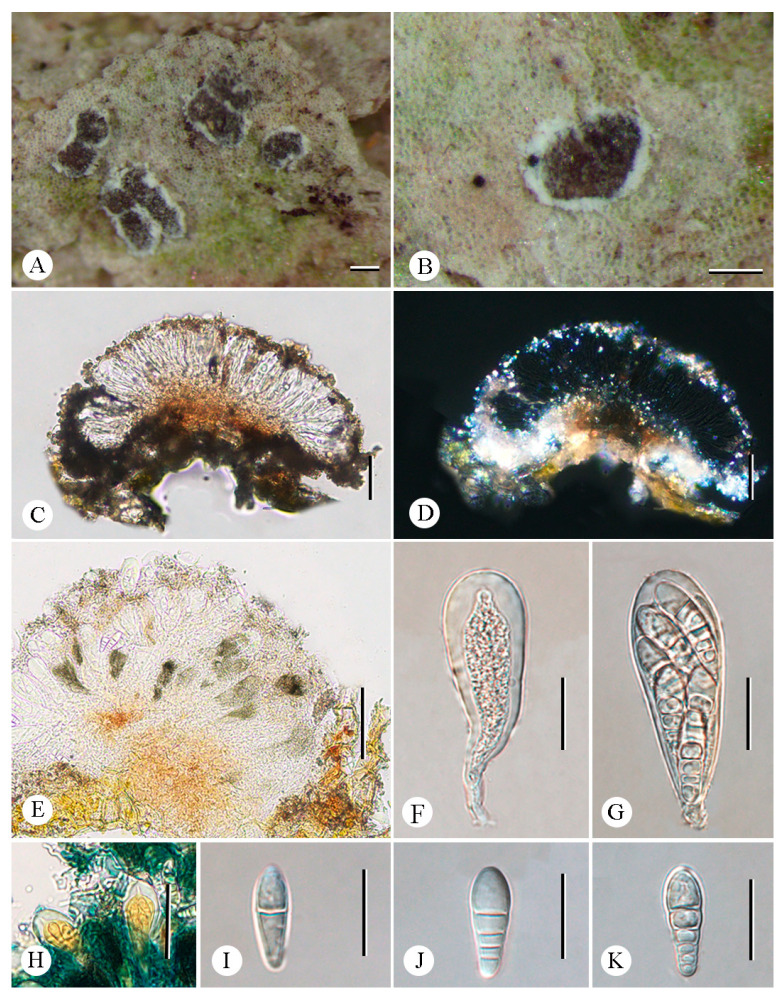
*Synarthonia inconspicua* (LCUF YN242231): (**A**,**B**) thallus with ascomata (**C**) apothecium section (**D**) apothecium section (in polarized light) showing distinct patches of calcium oxalate crystals (**E**) excipulum, K+ completely dissolving (**F**,**G**) asci, with eight ascospores (**H**) asci in 10% KOH stained with Lugol’s solution (**I**–**K**) ascospores in water. Scale bars: 2 mm (**A**); 1 mm (**B**); 50 µm (**C**–**E**); 20 µm (**F**, **G**); 50 µm (**H**); 20 µm (**I**–**K**).

***Synarthonia inconspicua*** (Stirt.) Van den Broeck and Ertz. (Figure 7).

Fungal Name: FN 825152.

**Basionym.** *Arthonia inconspicua* Stirt., Proceedings of the Philosophical Society of Glasgow 11: 319. 1879 [40].

**Description. **Thallus whitish to greenish-gray, smooth, continuous to cracked. Photobiont trentepohlioid, cells 9 µm in diam., rounded, usually in chains. Ascomata usually solitary, 0.25–0.6 × ca. 0.2 mm, rounded to lirellate, often forming irregular clusters, slightly elevated above thallus level, scattered more or less evenly over the thallus; disk heavily white pruinose in margin and thinner pruinose in the middle, light brown when pruina removed, flat to convex. Excipulum 16.5–21.5 µm wide, loosely intricate hyphae, inspersed with orange-brown granules which are K+ completely dissolving. Epihymenium 3.5–24.5 µm tall, and adspersed with orange-brown granules which are K+ completely dissolving. Hymenium 41.5–57.5 µm tall, hyaline, not inspersed, K/I+ blue. Paraphysoids loosely intricate around the asci. Hypothecium 17–34 µm-thick, yellowish brown, composed of loosely intricate hyphae, inspersed with orange granules which are K+ completely dissolving, K/I+ blue. Asci 49–65 × 17–24 µm, clavate, obovoid to ellipsoid or globose, stipitate, occasionally with a distinct ocular chamber, K/I−. Ascospores 17.5–24 × 6.5–8 µm, hyaline, with enlarged apical cell, oblong–ovoid, (1–)3–4(–5)-septate, spore ontogeny macrocephalic, unidirectional.

**Chemistry.** Thallus K-, C-, KC-, UV+. No substances were detected by TLC.

**Ecology and distribution.** Growing on exposed trees in tropical forests. Previously reported in the tropics of Tamil Nadu [40], India [41,42,43], Cuba, D.R. Congo, India, Madagascar, Netherlands Antilles, Rwanda, Sierra Leone, Tanzania, Uganda, and USA [17]. Newly reported in China.

**Additional specimens examined.** China. Yunnan Province: Mengla County, Mengla Fairyland, Menglazi Naturn Reserve, 21°42′44″ N, 101°22′32″ E, alt. 700 m, on bark, 19 December 2024, C.Y. Hao (LCUF YN242231).

**Notes.** The similar morphological species *Synarthonia albopruinosa* Van den Broeck and Ertz. differs from *S. inconspicua* in smaller ascospores with fewer septa (12.5–17.5 × 5–6.5 µm vs. 17.5–24 × 6.5–8 µm; (1–)2–3-septate vs. (1–)3–4(–5)-septate) [17]. Additionally, *S*. *fuscata* Van Den Broeck and Ertz. can be distinguished from *S. inconspicua* by the absence of pruina on the ascomata and the presence of a K/I+ blue ring-like structure in the asci [17]. In terms of molecular structure, newly generated sequences in this study of *S*. *inconspicua* were clustered with the published sequences of this species in the phylogenetic tree (Figure 1).

## 4. Discussion

Molecular analyses are indispensable for the classification and species identification of many lichen species within a genus due to their scarce morphological distinctions [44,45]. Employing morphological and phylogenetic analyses, this study identified three new species: *Arthonia yunnanensis*, *A. pararubella*, and *Eremothecella pruinocarpa*, and three new records for China: *A. rubella*, *E. calamicola*, and *Synarthonia inconspicua* from Yunnan Province, China. This study indicates the high species diversity of the family Arthoniaceae in Yunnan Province, China.

In the family Arthoniaceae, many taxa possess only phenotypic data but lack genotypic information, which has led to confusion regarding the taxonomic status; for example, *Eremothecella*. It needs to be pointed out that previous studies on this genus were mainly based on morphology, but this research provides the first molecular data for *Eremothecella*. Our molecular results also show that *Eremothecella* (*E. pruinocarpa* and *E. calamicola*) forms an independent clade within Arthoniaceae. Furthermore, to improve the clarity of generic relationships within the Arthoniaceae, sustained taxonomic investigations should be conducted in the future. Thorough surveys of the regional lichen flora will undoubtedly yield additional specimens for analysis. Empirical evidence indicates that in-depth research will uncover rarer species of Arthoniaceae, along with a considerable number of hitherto undescribed taxa.

## Figures and Tables

**Table 1:** Specimens of Arthoniaceae and the outgroup were used in the phylogenetic analyses.

Species	Specimen Voucher	GenBank Accession Number	
		mtSSU	RPB2
*Arthonia atra*	L10170	KY983978	KY983985
*Arthonia calcarea*	Thor 11/6a	KJ850974	KJ851105
*Arthonia calcarea*	Ertz 7545	EU704063	EU704027
*Arthonia didyma*	Ertz 7587	EU704047	EU704010
*Arthonia didyma*	Frisch 11/Se41	-	KJ851106
*Arthonia granithophila*	Frisch 10/Se74	KJ850981	KJ851107
*Arthonia graphidicola*	Frisch 10/Jp102	KJ850980	-
*Arthonia ilicina*	McCune 31067	KJ850982	-
*Arthonia incarnata*	Frisch 10/Jp94	KY983972	KY983980
*Arthonia incarnata*	Frisch 12/Jp289	KY983973	KY983981
*Arthonia incarnata*	Frisch 13/Jp215	KY983974	KY983982
*Arthonia maculiformis*	Wedin 9393b	-	KF707658
***Arthonia pararubella***	**YN242036**	**PV569153**	**PV590068**
***Arthonia pararubella***	**YN241783**	**PV569152**	-
*Arthonia physcidiicola*	Frisch 11/Ug318	KF707646	KF707657
*Arthonia radiata*	Frisch 10/Se29	KJ850968	KJ851108
*Arthonia radiata*	Frisch 11/Se25	KJ850969	KJ851109
*Arthonia rubella*	49024	MH308714	-
***Arthonia rubella***	**YN241343**	**PV569139**	-
***Arthonia rubella***	**YN241366**	**PV569140**	-
*Arthonia subfuscicola*	Thor 11/1	KJ850971	KJ851110
*Arthonia subfuscicola*	Frisch 11/Se15	KJ850972	KJ851111
*Arthonia thoriana*	Sanderson 2176	MG207687	-
*Arthonia thoriana*	Sanderson 2174	MG207685	-
***Arthonia yunnanensis***	**YN242413**	**PV569142**	**PV575682**
***Arthonia yunnanensis***	**YN242411**	**PV569141**	**PV575683**
*Arthonia atra*	L10170	KY983978	KY983985
*Arthonia calcarea*	Thor 11/6a	KJ850974	KJ851105
*Arthonia calcarea*	Ertz 7545	EU704063	EU704027
*Arthonia didyma*	Ertz 7587	EU704047	EU704010
*Arthonia didyma*	Frisch 11/Se41	-	KJ851106
*Arthonia granithophila*	Frisch 10/Se74	KJ850981	KJ851107
*Arthonia graphidicola*	Frisch 10/Jp102	KJ850980	-
*Arthonia ilicina*	McCune 31067	KJ850982	-
*Arthonia incarnata*	Frisch 10/Jp94	KY983972	KY983980
*Arthonia incarnata*	Frisch 12/Jp289	KY983973	KY983981
*Arthonia incarnata*	Frisch 13/Jp215	KY983974	KY983982
*Arthonia maculiformis*	Wedin 9393b	-	KF707658
***Arthonia pararubella***	**YN242036**	**PV569153**	**PV590068**
***Arthonia pararubella***	**YN241783**	**PV569152**	-
*Arthonia physcidiicola*	Frisch 11/Ug318	KF707646	KF707657
*Arthonia radiata*	Frisch 10/Se29	KJ850968	KJ851108
*Arthonia radiata*	Frisch 11/Se25	KJ850969	KJ851109
*Arthonia rubella*	49024	MH308714	-
***Arthonia rubella***	**YN241343**	**PV569139**	-
***Arthonia rubella***	**YN241366**	**PV569140**	-
*Arthonia subfuscicola*	Thor 11/1	KJ850971	KJ851110
*Arthonia subfuscicola*	Frisch 11/Se15	KJ850972	KJ851111
*Arthonia thoriana*	Sanderson 2176	MG207687	-
*Arthonia thoriana*	Sanderson 2174	MG207685	-
***Arthonia yunnanensis***	**YN242413**	**PV569142**	**PV575682**
***Arthonia yunnanensis***	**YN242411**	**PV569141**	**PV575683**
*Arthothelium galapagoense*	Ertz 11790	-	HQ454657
*Arthothelium galapagoense*	Ertz 11654	-	HQ454658
*Arthothelium norvegicum*	McCune 31061	-	KJ851114
*Arthothelium orbilliferum*	TRH-L-15449	KY983977	-
*Arthothelium punctatum*	KoLRI 044205	MF616614	-
*Arthothelium punctatum*	KoLRI 044206	MF616615	-
*Arthothelium ruanum*	KoLRI 038018	MF616609	MF616619
*Arthothelium ruanum*	KoLRI 038257	MF616610	MF616620
*Arthothelium ruanum*	KoLRI 038261	MF616611	MF616621
*Arthothelium ruanum*	KoLRI 038275	MF616612	-
*Arthothelium ruanum*	KoLRI 038275	MF616613	-
*Arthothelium ruanum*	Zimmerman 1117	GU327683	-
*Arthothelium* sp.	Joensson Guyana 10	KJ850957	KJ851094
*Arthothelium* sp.	Joensson Guyana 8	KJ850958	KJ851095
*Arthothelium spectabile*	TNS:Frisch12Jp179a	KP870144	KP870160
*Briancoppinsia cytospora*	Diederich 16849	JF830771	-
*Briancoppinsia cytospora*	Ertz 15244	JF830772	-
*Bryostigma apatetica*	Svensson 2017	KJ850992	KJ851125
*Bryostigma apatetica*	Svensson 1939	KJ850993	KJ851126
*Bryostigma apotheciorum*	Frisch 11/Se23	KJ850970	KJ851148
*Bryostigma biatoricola*	Thor 24350	KJ850990	KJ851149
*Bryostigma lapidicola*	Westberg Frisch 11/Se47	KJ850997	KJ851119
*Bryostigma lobariicola*	Frisch 10/Jp124	KJ851002	KJ851127
*Bryostigma lobariicola*	Frisch 10/Jp737	KJ851001	KJ851128
*Bryostigma molendoi*	Frisch 11/Se36	KJ851000	KJ851117
*Bryostigma molendoi*	Frisch 11/Se22	MH177777	MH177770
*Bryostigma muscigenum*	Thor 26206	KJ850991	KJ851124
*Bryostigma neglectula*	Frisch 10/Se91	KJ850989	KJ851118
*Bryostigma parietinaria*	Frisch 17/No25	MH177778	MH177771
*Bryostigma peltigerina*	Westberg Frisch 11/Se46	KJ850998	KJ851122
*Bryostigma phaeophysciae*	Ertz 11/2	-	KJ851112
*Bryostigma* sp.	Svensson 2324	KJ851019	KJ851123
*Bryostigma* sp.	SK L06	MH510022	MH510019
*Bryostigma* sp.	Svensson 2148	KJ850995	KJ851120
*Bryostigma* sp.	SK L04	MH510023	MH510020
*Bryostigma stereocaulina*	Westberg Frisch 11/Se48	KJ850999	-
*Bryostigma toensbergii*	Frisch N4-2-Pa4-1	MH177775	-
*Bryostigma toensbergii*	Frisch N4-2-Pa6-1	MH177776	-
*Coniocarpon* aff. *cinnabarinum*	Frisch 11/Ug3	KJ850978	KJ851102
*Coniocarpon cinnabarinum*	Johnsen111003	KJ850976	KJ851103
*Coniocarpon cinnabarinum*	Frisch11Ug296	KP870158	KP870170
*Coniocarpon fallax*	LD:L10075	KJ850979	KJ851101
*Coniocarpon rubrocinctum*	Nelsen 4010	GU327684	-
*Crypthonia* aff. *vandenboomii*	Frisch 11/Ug21	KJ850960	KJ851085
*Crypthonia palaeotropica*	Frisch 11/Ug457	KJ850961	KJ851084
*Crypthonia palaeotropica*	Frisch11Ug26B	KP870145	KP870161
*Cryptophaea phaeospora*	Van den Broeck 5809	KX077541	-
*Cryptophaea phaeospora*	Van den Broeck 5964	KX077540	-
*Cryptothecia assimilis*	Lumbsch 198151	GU327688	-
*Cryptothecia austrocoreana*	KoLRI No. 041892	MF769374	-
*Cryptothecia austrocoreana*	KoLRI No. 044721	MF769375	-
*Cryptothecia punctosorediata*	F:Nelsen 4038	JX046450	-
*Cryptothecia* sp.	Frisch 11/Ug39	KJ850954	KJ851086
*Cryptothecia* sp.	Frisch 11/Ug18	KJ850955	KJ851092
*Cryptothecia* sp.	Frisch 11/Ug194	KJ850956	KJ851093
*Cryptothecia subnidulans*	v.d.Boom 40613	KJ850952	KJ851087
*Cryptothecia subnidulans*	Joensson Guyana 6a	KJ850953	KJ851088
*Diarthonis spadicea*	Thor 26200	-	KJ851116
*Diarthonis spadicea*	Frisch 11/Se31	-	KJ851115
***Eremothecella pruinocarpa***	**YN242390-1**	**PV569148**	**PV575678**
***Eremothecella pruinocarpa***	**YN242384**	**PV569149**	**PV575679**
***Eremothecella pruinocarpa***	**YN242386-2**	**PV569150**	**PV575680**
***Eremothecella pruinocarpa***	**YN242387-2**	**PV569151**	**PV575681**
***Eremothecella calamicola***	**YN242386-1**	**PV569146**	**-**
***Eremothecella calamicola***	**YN242387-1**	**PV569147**	**-**
***Eremothecella calamicola***	**YN242388**	**PV569144**	**-**
***Eremothecella calamicola***	**YN242390-2**	**PV569145**	**PV575677**
*Glomerulophoron mauritiae*	BR Ertz19164	KP870153	KP870167
*Herpothallon inopinatum*	Rudolphi 12	KJ850964	KJ851099
*Herpothallon kigeziense*	Frisch 11/Ug26	KF707644	KF707654
*Herpothallon rubrocinctum*	Rudolphi 5	KF707643	KF707655
*Herpothallon rubrocinctum*	Nelsen 4006	GU327693	-
*Herpothallon* sp.	Frisch 11/Ug401	KF707645	KF707653
*Inoderma afromontanum*	Frisch 11/Ug164	KJ850963	KJ851090
*Inoderma byssaceum*	Thor 25952	KJ850962	KJ851089
*Inoderma byssaceum*	Lif 186	-	KJ851091
*Inoderma nipponicum*	TNS:Frisch12Jp227	KP870146	KP870162
*Inoderma nipponicum*	TNS:Frisch13Jp1	KP870147	KP870163
*Inoderma sorediatum*	Kukwa 15631 & Lubek	MG207690	-
*Inoderma subabietinum*	Ertz16885	KP870150	KP870164
*Leprantha cinereopruinosa*	Kukwa 17127 & Lubek	MG207692	-
*lnoderma sorediatum*	Kukwa 15630 & Lubek	MG207689	-
*Melarthonis piceae*	Thor 25995	KJ851016	-
*Myriostigma candidum*	Ertz 9260	EU704052	EU704015
*Myriostigma candidum*	Frisch 11/Ug125	KJ850959	KJ851096
*Myriostigma miniatum*	Silva T2A29	KP843606	-
*Naevia aff punctiformis*	Thor 24702	KJ850975	-
*Naevia dispersa*	UPSC 2583	AY571383	-
*Naevia pinastri*	MFLU 17-3497	MN842780	-
*Naevia punctiformis*	Thor 26158	KJ850973	KJ851113
*Pachnolepia pruinata*	Tehler 9139	-	HQ454650
*Pachnolepia pruinata*	Frisch 11/Se34	KJ850967	KJ851098
*Reichlingia leopoldii*	Ertz 13293	JF830773	HQ454722
*Reichlingia leopoldii*	Ertz 13294	JF830774	HQ454723
*Reichlingia syncesioides*	Frisch 11/Ug14	KF707651	KF707656
*Reichlingia zwackhii*	Thor 11/3	KF707652	KF707662
*Reichlingia zwackhii*	Ertz 10928	-	HQ454655
*Snippocia nivea*	Ertz 17437	MG207695	-
*Snippocia nivea*	Sanderson 2180	MG207694	-
*Snippocia nivea*	Sanderson 2179	MG207693	-
*Sporodophoron cretaceum*	Thor27720	KP870159	-
*Sporodophoron gossypinum*	TNS:Frisch12Jp186	KP870154	KP870168
*Sporodophoron gossypinum*	TNS:Frisch12Jp197	KP870156	-
*Sporodophoron primorskiense*	TNS:Y.Ohmura10607	LC086299	-
*Sporodophoron primorskiense*	TNS:Ohmura10509	KP870157	KP870169
*Stirtonia neotropica*	Cáceres & Aptroot 11112	KP843611	-
*Stirtonia* sp.	Frisch 11/Ug325	KJ850965	-
*Synarthonia albopruinosa*	VDB 6086	MH251873	-
*Synarthonia aurantiacopruinosa*	VDB 5764	MH251874	-
*Synarthonia fuscata*	VDB 6101	MH251875	-
*Synarthonia inconspicua*	VDB 7013B	MH251881	-
*Synarthonia inconspicua*	ERTZ 19739A	MH251879	-
***Synarthonia inconspicua***	**YN242231**	**PV569143**	**-**
*Synarthonia josephiana*	ERTZ 19739B	MH251876	-
*Synarthonia muriformis*	Frisch 11/Ug41	KJ851025	-
*Synarthonia muriformis*	ERTZ 19344	MH251877	-
*Synarthonia ochracea*	VDB 6653	MH251884	-
*Synarthonia pilosella*	ERTZ 7808	MH251883	-
*Tylophoron galapagoense*	Bungartz 8749	JF830776	-
*Tylophoron galapagoense*	Bungartz 8750	JF830777	-
*Tylophoron hibernicum*	Diederich 16335	JF830779	-
*Tylophoron hibernicum*	Frisch 11/Ug220	KJ850966	KJ851097
*Tylophoron moderatum*	Ertz 14504	JF830780	-
*Tylophoron stalactiticum*	Ertz 10880	JF830781	-

Notes: Newly generated sequences are shown in bold.

## Data Availability

Publicly available datasets were analyzed in this study. These data can be found at http://www.ncbi.nlm.nih.gov/ (accessed on 25 April 2025).

## References

[1] Reichenbach H.T.L., Her barienbuch D. (1841). Der Deutsche Botaniker.

[2] Tehler A. (1990). A new approach to the phylogeny of *Euascomycetes* with a cladistic outline of Arthoniales focussing on Roccellaceae. Can. J. Bot..

[3] Grube M. (1998). Classification and Phylogeny in the Arthoniales (*Lichenized ascomycetes*). Bryologist.

[4] Frisch A., Thor G., Moon K.H., Ohmura Y. (2017). *Arthonia incarnata* (Arthoniaceae), a rare and poorly known old-growth forest lichen new to Asia. Nord. J. Bot..

[5] Thiyagaraja V., Lücking R., Ertz D., Wanasinghe D.N., Karunarathna S.C., Camporesi E., Hyde K.D. (2020). Evolution of non-lichenized, saprotrophic species of Arthonia (Ascomycota, Arthoniales) and resurrection of Naevia, with notes on Mycoporum. Fungal Divers..

[6] Frisch A., Thor G., Ertz D., Grube M. (2014). The Arthonialean challenge: Restructuring Arthoniaceae. Taxon.

[7] Aptroot A., Cáceres M.E.D.S., Santos L.A.D. (2024). The taxonomy of sterile *Arthoniaceae* from Brazil: White crusts on overhanging tropical trees can be named. Lichenologist.

[8] Lücking R., Hodkinson B.P., Leavitt S.D. (2017). The 2016 classification of lichenized fungi in the Ascomycota and Basidiomycota—Approaching one thousand genera. Bryologist.

[9] Sundin R., Thor G., Frisch A. (2012). A literature review of Arthonia s. lat. Bibl. Lichenol..

[10] Grube M., Matzer M., Hafellner J. (1995). A Preliminary Account of the Lichenicolous *Arthonia* Species with Reddish, K+ Reactive Pigments. Lichenologist.

[11] Cannon P., Ertz D., Frisch A., Aptroot A., Chambers S., Coppins B., Sanderson N., Simkin J., Wolseley P. (2020). Arthoniales: Arthoniaceae, including the genera Arthonia, Arthothelium, Briancoppinsia, Bryostigma, Coniocarpon, Diarthonis, Inoderma, Naevia, Pachnolepia, Reichlingia, Snippocia, Sporodophoron, Synarthonia and Tylophoron. Revis. Br. Ir. Lichens.

[12] Sydow H. (1917). Beitrag zur Kenntnis der Pilzflora der Philippinen-Inseln. Ann. Mycol..

[13] Sérusiaux E. (1992). Reinstatement of the lichenized genus Eremothecella Sydow. Syst. Ascomycetum.

[14] Lücking R. (2008). Foliicolous Lichenized Fungi.

[15] Santesson R. (1952). Foliicolous Lichens 1. A Revision of the Taxonomy of the Obligately Foliicolous Lichenized Fungi.

[16] Jagadeesh Ram T.A.M., Sinha G.P. (2019). New species and first records of Eremothecella (Arthoniales) from the Andaman and Nicobar Islands, India. Lichenologist.

[17] Van Den Broeck D., Frisch A., Razafindrahaja T., Van De Vijver B., Ertz D. (2018). Phylogenetic position of Synarthonia (lichenized Ascomycota, *Arthoniaceae*), with the description of six new species. Plant Ecol. Evol..

[18] Joseph S., Sinha G.P. (2015). Contributions to the genus *Synarthonia* (lichenized Ascomycota, *Arthoniaceae*). Lichenologist.

[19] Ertz D., Aptroot A., Sanderson N., Coppins B., Van Den Broeck D., Diederich P. (2020). A new species of *Synarthonia* from Luxembourg, and a new combination in the genus *Reichlingia* (*Arthoniaceae*). Lichenologist.

[20] Yao Z., Jiang S., Jia Z. (2021). Mazosia weii sp. nov. (Roccellaceae) from China, a new species supported by molecular data. Bryologist.

[21] Cui C., Dou M., Jiang S., Jia Z. (2024). The Lichen Genus Letrouitia (Brigantiaeaceae, Ascomycota) in China. Diversity.

[22] Orange A., James P.W., White F.J. (2001). Microchemical Methods for the Identification of Lichens.

[23] Zoller S., Scheidegger C., Sperisen C. (1999). Pcr Primers for the Amplification of Mitochondrial Small Subunit Ribosomal DNA of Lichen-forming Ascomycetes. Lichenologist.

[24] Liu Y.J., Whelen S., Hall B.D. (1999). Phylogenetic relationships among ascomycetes: Evidence from an RNA polymerse II subunit. Mol. Biol. Evol..

[25] Shi K., Jia Z., Zhao X. (2023). A New Species and Two New Records of the Lichen Genus Fissurina from China. Diversity.

[26] Castresana J. (2000). Selection of conserved blocks from multiple alignments for their use in phylogenetic analysis. Mol. Biol. Evol..

[27] Miller M.A., Pfeiffer W., Schwartz T. Creating the CIPRES Science Gateway for inference of large phylogenetic trees. Proceedings of the 2010 Gateway Computing Environments Workshop (GCE).

[28] Stamatakis A. (2014). RAxML version 8: A tool for phylogenetic analysis and post-analysis of large phylogenies. Bioinformatics.

[29] Lanfear R., Frandsen P.B., Wright A.M., Senfeld T., Calcott B. (2017). PartitionFinder 2: New Methods for Selecting Partitioned Models of Evolution for Molecular and Morphological Phylogenetic Analyses. Mol. Biol. Evol..

[30] Ronquist F., Teslenko M., Van Der Mark P., Ayres D.L., Darling A., Höhna S., Larget B., Liu L., Suchard M.A., Huelsenbeck J.P. (2012). MrBayes 3.2: Efficient Bayesian Phylogenetic Inference and Model Choice Across a Large Model Space. Syst. Biol..

[31] Fée A.L.A. (1825). Essai sur les Cryptogames des Écorces Exotiques Officinales.

[32] Nylander W. (1856). Synopsis du genre Arthonia. Mémoires Société Impériale Sci. Nat. Cherbg..

[33] Willey H. (1890). A Synopsis of the Genus Arthonia.

[34] Seavey F., Seavey J. (2012). Caloplaca lecanorae (Teloschistaceae), a new lichenicolous lichen and several additions to the North American lichenized mycota from Everglades National Park. Bryologist.

[35] Vainio E.A. (1921). Lichenes insularum Philippinarum, III. Ann. Acad. Sci. Fenn. Ser. A.

[36] Wang W.C., Wei J.C. (2018). *Arthonia*, *Byssoloma*, *Calenia*, *Chroodiscus*, *Coenogonium*, *Eremothecella*, and *Semigyalecta* spp. new to China. Mycotaxon.

[37] Lücking R., Streimann H., Elix J.A. (2001). Further records of foliicolous lichens and lichenicolous fungi from Australasia, with an updated checklist for continental Australia. Lichenologist.

[38] Esslinger T.L. (2016). A Cumulative Checklist for the Lichen-Forming, Lichenicolous and Allied Fungi of the Continental United States and Canada, Version 21. Opusc. Philolichenum.

[39] Diederich P., Common R.S., Braun U., Heuchert B., Millanes A., Suija A., Ertz D. (2019). Lichenicolous fungi from Florida growing on Graphidales. Plant Fungal Syst..

[40] Stirton J. (1879). New and rare lichens from India and the Himalayas. Proc. Philos. Soc. Glasg..

[41] Nakanishi M. (1981). Notes on lichen species of Graphis of the Yaeyama Islands, Japan. Hikobia Suppl..

[42] Müller J., Durand T.H., Pittier H. (1891). Lichenes. Primitiae Florae Costaricensis.

[43] Dodge C.W. (1953). Some lichens of tropical Africa. Ann. Mo. Bot. Gard..

[44] Jiang S.H., Lücking R., Xavier-Leite A.B., Cáceres M.E.S., Aptroot A., Portilla C.V., Wei J.C. (2020). Reallocation of foliicolous species of the genus Strigula into six genera (lichenized Ascomycota, Dothideomycetes, Strigulaceae). Fungal Divers..

[45] Lebreton E., Ertz D., Lücking R., Aptroot A., Carriconde F., Ah-Peng C., Huang J.-P., Chen K.-H., Stenger P.-L., Cáceres M.E.D.S. (2025). Global phylogeny of the family Gomphillaceae (Ascomycota, Graphidales) sheds light on the origin, diversification and endemism in foliicolous lineages. IMA Fungus.

